# Eating Disorder Behaviors Are Increasing: Findings from Two Sequential Community Surveys in South Australia

**DOI:** 10.1371/journal.pone.0001541

**Published:** 2008-02-06

**Authors:** Phillipa J. Hay, Jonathan Mond, Petra Buttner, Anita Darby

**Affiliations:** 1 School of Medicine, University of Western Sydney, Sydney, Australia; 2 School of Psychological Science, La Trobe University, Melbourne, Victoria, Australia; 3 School Public Health, Tropical Medicine and Rehabilitation Science, James Cook University, Townsville, Queensland, Australia; 4 Discipline of Psychiatry, School of Medicine, James Cook University, Townsville, Queensland, Australia; Office of the WHO Representative in Sudan, Sudan

## Abstract

**Background:**

Evidence for an increase in the prevalence of eating disorders is inconsistent. Our aim was to determine change in the population point prevalence of eating disorder behaviors over a 10-year period.

**Methodology/Principal Findings:**

Eating disorder behaviors were assessed in consecutive general population surveys of men and women conducted in 1995 (n = 3001, 72% respondents) and 2005 (n = 3047, 63.1% respondents). Participants were randomly sampled from households in rural and metropolitan South Australia. There was a significant (all p<0.01) and over two-fold increase in the prevalence of binge eating, purging (self-induced vomiting and/or laxative or diuretic misuse) and strict dieting or fasting for weight or shape control among both genders. The most common diagnosis in 2005 was either binge eating disorder or other “eating disorders not otherwise specified” (EDNOS; n = 119, 4.2%).

**Conclusions/Significance:**

In this population sample the point prevalence of eating disorder behaviors increased over the past decade. Cases of anorexia nervosa and bulimia nervosa, as currently defined, remain uncommon.

## Introduction

There is continued debate over the question of whether the prevalence of eating disorders is increasing. Estimates of the prevalence of psychiatric disorders rest on accurate recognition and delineation of disorders in classification schemes, and the development of methods for community-based epidemiological studies, such as the Epidemiologic Catchment Areas study in 1980 [1]. While anorexia nervosa was the first eating disorder to be recognised, with the 19^th^ century reports of Gull [2] and Lesague [3], bulimia nervosa, and binge eating disorder were not described until nearly a century later [4]–[6]. It is now agreed that the first estimates of general population point prevalence of eating disorders likely overestimated bulimia nervosa and the ‘second generation’ of studies (for example that of Bushnell *et al* [7]) had been consistent in finding bulimia nervosa to occur in around 1% of young western women and that partial eating disorder syndromes or eating disorder not otherwise specified (EDNOS), [6] occur in between 2 and 5% of young women [8]. In contrast anorexia nervosa likely occurs in less than 0.5% of young women and is uncommon in the general population, for example as found by Aalto-Setala and colleagues [9].

Accurate incidence studies have been more difficult to complete but cohort and clinical incidence studies [7], [10] have supported an increase in the incidence of bulimia nervosa over the 10 years (i.e. the 1980s) following its recognition, with one study suggesting this has since reached a peak [11]. Sequential population surveys have been problematic and variable in regard to case definition and ascertainment, but those that have been done have not reported an increase since the late 1980s [12]. In contrast to bulimia nervosa, clinical incidence studies of anorexia nervosa have accessed likely cases more through medical records of patients presenting with its physical complications, such as infertility, and unexplained weight loss [13], [14]. A systematic review [15] of cumulative incidence studies reported an estimated mean yearly incidence in the general population of 8 cases per 100,000 with a likely increase in the incidence of anorexia nervosa in young women in the last century up to the 1970s. The estimated incidence of bulimia nervosa was 12 cases per 100,000 per year [15].

Most recently a study of time trends in general practice databases in the UK [16] reported an initial increase and then decrease in bulimia nervosa incidence. A similar study of time trends in primary care from the Netherlands [17] found a decrease in incidence of bulimia nervosa. In addition, a US study involving consecutive surveys of college students [18] from 1982, 1992, and 2002, suggest that incidence and/or prevalence of bulimia nervosa and related disorders may be decreasing over the 20 year period. Findings from national surveys indicate that the 12-month or current prevalence of eating disorders in recent community surveys in North America [19], New Zealand [20] and Europe [21] may vary by more than four fold, and in North America Hudson and colleagues [19] found cohort effects supporting a putative increase in prevalence of eating disorders over time and comparatively high rates (2.1%) of binge eating in both men (1.7%) and women (2.5%). The present study was planned to investigate changes, if any, in the community prevalence of eating disorder behaviors over a 10 year period in South Australia and to compare the findings with these international studies conducted over the past decade.

## Methods

### Design

Two independent cross-sectional single stage interview based surveys were conducted a decade apart. Both surveys were embedded in the respective year Health Omnibus Survey, under the auspices of the South Australian Health Commission. The interviews were conducted by Harrison Health Research [8], [22].

### Sample selection and interview procedures

The sample selection and interview procedures were the same for each survey. Samples were selected from both metropolitan and rural areas. For the metropolitan sample in 1995 320, and in 2005 386 “collectors' districts” were selected from those used by the Australian Bureau of Statistics in the 1991 and 2001 census respectively. For the country samples, all towns of 10,000 or more in population size and a selection of towns of at least 1,000 people were surveyed. The collectors' districts were chosen according to their probability of selection proportional to size. Within each collector's district a starting point was randomly selected. From this starting point, using a pre-determined process based on a “skip” pattern of every fourth household, 10 dwellings were chosen. Only one interview was conducted per household or dwelling, and, where more than one resident was aged over 15 years, the respondent was the person whose birthday was last. The sample was a non-replacement sample, and up to six separate visits were made to interview the person chosen to take part.

The interview was piloted during February 1995 and August 2005, with 50 interviews. No formal reliability study was done, but 5% in 1995 and 10% in 2005 of each interviewer's work was selected at random, and the respondents re-contacted and a number of questions were asked of them, to ensure they had been interviewed as reported. Interviews were conducted from March until the second week of May 1995 and September through to 31^st^ December 2005.

### The interview

The structured, respondent-based interview, comprised a range of health-related and demographic questions, including present height and weight. The eating disorder behavior questions were written by the first author (PH), and were modelled on related questions used in the investigator-based interview, the Eating Disorder Examination (EDE; 23). (The EDE, is however a much more detailed and comprehensive assessment of symptoms, particularly binge eating.) The questions were embedded towards the end of the interview. Three eating disorder behaviors were assessed, namely binge eating, purging and strict dieting or fasting. Current regular use of these behaviors was defined as the behavior occurring at least weekly over the three months prior to the interview. In order to assess diagnostic criteria for bulimia nervosa and related EDNOS and an estimate of burden, in 2005 two additional questions were added, namely one assessing importance of weight and shape and one assessing ‘days out of role’ [24]. The former was modeled on the Importance of Weight and Importance of Shape questions employed in the EDE. Because these questions were not asked in the 1995 survey, it was possible to consider 1) change in the prevalence of eating disorder behaviors over time and 2) prevalence of diagnoses in 2005, but not change in prevalence of diagnoses over time. Body mass index (BMI; kg/m^2^) was calculated from self-reported weight and height.

The specific questions relating to eating disorders in the survey were: (i) *I would now like to ask you about episodes of overeating that you may have had recently. By overeating, or binge eating, I mean eating an unusually large amount of food in one go and at the time feeling that your eating was out of control, [that is you could not prevent yourself from overeating, or that you could not stop eating once you had started]. Over the past three months how often have you overeaten in the way I have described?* Responses were made from a 4-point list of ‘not at all’, ‘less than weekly’, ‘once a week’ and ‘two or more times a week’; (ii) *The next questions are about various weight-control methods some people use. Over the past three months have you regularly used, that is at least ONCE A WEEK, used any of the following: laxatives, diuretics (water tablets), made yourself sick, in order to control your shape or weight?* Responses were either ‘yes’ or ‘no’; (iii) *Over the past three months have you regularly e.g. at least once weekly, or recurrently during the three months, done any of the following: gone on a very strict diet, or eaten hardly anything at all for a time, in order to control your shape or weight?* Responses were either ‘yes’ or ‘no’; (iv) *In the past three months has your weight and/or your shape influenced how you think about (judge) yourself as a person? E.g. has it been a really important issue to you/to your self-confidence?* Responses were on a 6-point scale from ‘not at all’ to ‘extremely (the most important thing for you)’ with a cut-off of ≥4 to indicate at least moderate importance; and (v) *During the past four weeks, on how many days (approximately), if any, were you unable to complete your work, study or household responsibilities because of any problem with your physical or emotional health?* The number of days between 0 and 28 was recorded.

### Statistics

Data were weighted by the inverse of the individual's probability of selection, then re-weighted to benchmarks derived from the Estimated Resident Populations at 30th June 1994, by age, sex and Local Government Area, from the Australian Bureau of Statistics (Catalogue No 3204.4). The stratified cluster sampling approach was taken into account during the entire statistical analysis.

Numerical data were presented as mean values and standard deviations (SD). T-tests and chi–squared tests as appropriate were used to test for bivariate differences between the 1995 and 2005 surveys. Estimated prevalence of eating disorders were accompanied by 95%-confidence intervals (95%-CI). Multivariable logistic regression analyses were conducted to determine the odds of reporting each eating disorder behavior as a function of the year of study (1995 and 2005) controlling for the baseline values of potential confounders, namely, age, gender, body mass index, country of birth, marital status, level of education, and income. Multivariate analyses were stratified by gender. Results of multivariate analyses are presented as odds-ratios and 95%-confidence intervals. A significance level of 0.05 was employed for all tests. Analyses were conducted using the SPSS for Windows version 14 and the survey commands of STATA release 8.

### Ethics

All subjects in the study gave verbal informed consent to their participation and the study was approved as ethical by the Government of South Australia Department of Health. All participants received written information about the survey prior to consent being obtained. Written consent was deemed impractical in this large low risk survey by the Department, verbal consent was obtained by the interviewers and audited by the Department, and the oral informed consent process was approved by the research ethics committee of the Department.

## Results

### The samples

Demographic and other details of the sample and respondents are shown in Table 1. There was a decrease in the overall response rate, with numbers of refusals to participate increasing.

**Table 1:** Comparative demographic and socio-economic data for the 1995 and 2005 surveys.

	1995 survey (n = 3001)	2005 survey (n = 3047)	p-value
% Response rate	71.5%	60.9%	<0.001
% Reasons for non-response	(n = 1199)	(n = 1953)	
Refusal	45.4%	51.8%	
Failure to establish contact	33.4%	28.8%	
Vacant house	11.3%	8.9%	
Lack of fluency in English	4.8%	3.6%	
Not at home during survey period	4.1%	3.3%	
Illness or other incapacity	1.2%	3.7%	
Mean age (SD) [years]	43.4 (19.2)	45.1 (24.5)	0.002
Mean body mass index (SD) [kg/m^2^]	25.1 (5.6) (n = 2765)	26.0 (6.1) (n = 2802)	<0.001
% Female	50.8%	51.0%	0.902
% Metropolitan based	69.3%	70.1%	0.775
% Born in Australia	76.5%	77.6%	0.418
% Married or de-facto	62.0%	62.4%	0.804
% With highest educational attainment graduate diploma or degree or higher	20.7%	27.1%	<0.001
% With yearly household income greater than $AUD 50,000	20.0% (n = 2669)	50.0% (n = 2698)	<0.001

Excepting response rates, all results were adjusted for cluster sampling.

SD = standard deviation

Significant differences between the samples were observed with respect to age (p = 0.002), BMI (p<0.001), annual household income (p<0.001) and educational attainment (p<0.001). There were no significant differences on any of the other socio-demographic variables assessed.

### Prevalence of eating disorder behaviors

Results of multivariable logistic regression analyses showed that there were significant increases in the current regular use of all three eating disorder behaviors in both men and women (Table 2). In comparison to 1995, participants were 2.4 times more likely to report binge eating in 2005 (p<0.001).

**Table 2:** Comparative distribution over time of regular current^1^ eating disorder behaviors.

	1995 survey	2005 survey	Odds-ratio 95%-CI^5^	p-value
*All participants*	(n = 3001)	(n = 3047)		
Binge eating^2^	96 (3.1%)	205 (7.2%)	2.4 [1.6, 3.7]	<0.001
Purging^3^	24 (0.7%)	54 (1.5%)	2.9 [1.4, 5.8]	= 0.003
Strict dieting or fasting^4^	48 (1.6%)	129 (4.6%)	2.6 [1.7, 3.9]	<0.001
*Male participants*	(n = 1216)	(n = 1290)		
Binge eating	36 (3.1%)	77 (7.8%)	2.1 [1.2, 3.5]	= 0.007
Purging	0 (0%)	14 (1.0%)	NA^6^	<0.001
Strict dieting or fasting	8 (0.6%)	44 (3.9%)	5.1 [2.1, 12.0]	<0.001
*Female participants*	(n = 1785)	(n = 1757)		
Binge eating	60 (3.2%)	128 (7.5%)	2.5 [1.6, 3.9]	<0.001
Purging	24 (1.3%)	40 (2.1%)	1.5 [0.85, 2.8]	= 0.152
Strict dieting or fasting	40 (2.5%)	85 (5.2%)	2.0 [1.3, 3.2]	= 0.002

All results were adjusted for the cluster sampling approach.

1 Current regular use of these behaviors was defined as the behavior occurring at least weekly over the three months prior to the interview;

2 Binge eating was described as episodes of overeating, namely eating an unusually large amount of food in one go and at the time feeling that the eating was out of control, (i.e. it could not be prevented or stopped);

3 Purging was described as a weight control method comprising the use of laxatives, diuretics (water tablets), or self-induced vomiting;

4 Strict dieting was described as “going on a very strict diet”, and fasting as “eating hardly anything at all for a time”, both for the purpose of weight or shape control;

5 Multivariate adjusted odds ratio and 95%-confidence interval; adjusted for age, gender, BMI, country of birth, education and income level as relevant;

6 Because of zero frequency of purging in 1995 males an adjusted odds ratio could not be calculated, and a bivariate chi-squared test adjusted for cluster sampling was conducted.

Age increased not only from one cross-section to the next, but also, as shown on Table 3 in the subgroups of individuals reporting purging (p = 0.025) and strict dieting or fasting (p<0.001). Similarly, amongst the participants who reported strict dieting or fasting behavior mean body mass index increased from 25.2 (SD 7.1) in 1995 to 28.4 (7.0) in 2005 (p = 0.011). Within the sub-groups of participants with reported eating disorder behaviors the percentage of participants living in metropolitan areas did not change between 1995 and 2005 (binge eating: p = 0.955; purging: p = 0.241; strict dieting or fasting: p = 0.940).

**Table 3:** Age distributions of participants with regular current^1^ eating disorder behaviours.

	Binge eating^2^		Purging^3^		Strict dieting or fasting^4^	
Age (years)	1995 (n = 96)	2005 (n = 205)	1995 (n = 24)	2005 (n = 54)	1995 (n = 48)	2005 (n = 129)
15–24	21.9%	29.3%	19.8%	13.6%	45.2%	20.0%
25–34	34.3%	21.3%	13.5%	9.0%	29.2%	26.5%
35–44	21.7%	18.4%	43.9%	21.2%	15.2%	17.1%
45–54	18.0%	17.4%	11.0%	28.6%	8.7%	21.4%
55–64	1.7%	7.4%	6.7%	7.5%	0%	9.7%
> = 65	2.3%	6.2%	5.2%	20.1%	1.7%	5.3%
Mean (SD) 5	34.4 (13.1)	36.4 (19.8)	38.5 (14.6)	48.0 (21.7)	27.9 (10.7)	38.6 (19.8)
p-value	P = 0.265		P = 0.025		P<0.001	

All results were adjusted for the cluster sampling approach.

1 Current regular use of these behaviors was defined as the behavior occurring at least weekly over the three months prior to the interview.

2 Binge eating was described as episodes of overeating, namely eating an unusually large amount of food in one go and at the time feeling that the eating was out of control, (i.e. it could not be prevented or stopped).

3 Purging was described as a weight control method comprising the use of laxatives, diuretics (water tablets), or self-induced vomiting.

4 Strict dieting was described as “going on a very strict diet”, and fasting as “eating hardly anything at all for a time”, both for the purpose of weight or shape control.

5 SD = standard deviation

### Estimated prevalence and burden of eating disorders

The 1995 study was not designed to evaluate prevalence of eating disorders, since the question about shape and weight concerns was not asked (i.e. there was no assessment of the “undue influence of weight or shape on self-evaluation”.) In 2005, using the Oxford criteria of current (over the preceding three months) *weekly* frequency of binge eating and extreme weight control behaviors [23], there were 24 (0.9%, 95% CI 0.5%–1.4%) participants with bulimia nervosa (majority n = 20 84% female), 6 (all female) of purging type, and 70 (2.3%, 95% CI 1.7%–2.9%) subjects with binge eating disorder (majority n = 47, 67% female) broadly defined to comprise those who described regular (on average weekly) episodes of binge eating, who did not use extreme weight-reducing behaviors regularly and who reported weight and/or shape concerns of at least moderate importance, and 49 (1.9%, 95% CI 1.3%–2.5%) other EDNOS cases (majority n = 34 69% female) where participants reported weight and/or shape concerns of at least moderate importance in association with regular extreme weight control behaviors (15 purging type). Although the study was not designed to assess all criteria for anorexia nervosa, five individuals (0.3%, 95% CI 0.02%–0.5%) were identified with BMI≤17.5 and moderate or more weight and/or shape concerns. Four of these five were women. None of these five possible cases however reported any current weight control behaviors and/or binge eating.

‘Days out of role’ were more frequent for the 143 with a current eating disorder (1 day: 4.7%, 2 days: 5.9%, 3 days: 6.1%, 4 days: 7.3%, 5 or more days: 19.8%) compared to the 2904 participants without an eating disorder (1 day: 4.0%, 2 days: 4.1%, 3 days: 2.1%, 4 days: 1.7%, 5 or more days: 9.5%) (p<0.001).

## Discussion

Between 1995 and 2005 there was a significant and over two-fold increase in the point prevalence of binge eating, purging (self-induced vomiting and/or laxative or diuretic misuse) and strict dieting or fasting for weight or shape control. These behaviors increased in both men and women. The most common diagnosis in 2005 was EDNOS, inclusive of binge eating disorder.

The present study was limited in that assessment was only at two time points and weight and shape concerns were not assessed in 1995. In addition, there was no assessment (unlike the Fichter *et al* [21] study) of subjective bulimic episodes (where the quantity eating during the binge is not objectively large) and thus EDNOS where sufferers have subjective bulimic episodes and concerns without extreme regular weight control behaviors would not have been detected. There was also no assessment of excessive exercise. It would also be desirable to have included assessment of lifetime as well as current prevalence rates. Notable strengths of the present study were the standardized and rigorous survey methods, including random selection and interview assessment, the good response rates, wide age range and inclusion of both genders. Previous studies have been limited by the use of general practice databases [16], [17] or student samples [18], [25].

As in other community surveys, anorexia nervosa appeared to be rare and in this survey no potential cases were detected. However, there were five participants with a BMI below 18 who reported extreme weight and shape concerns, but who denied any extreme weight control behaviors or attempting to lose weight. It is possible that current diagnostic criteria for anorexia nervosa [6], [23] do not capture people in the community who have the problem, but who have yet to acknowledge key symptoms such as self-imposed dietary restriction and fear of weight gain. In addition, there is evidence that individuals with anorexia nervosa and partial-syndrome variants of anorexia nervosa may be over-represented among non-respondents in general population surveys” [26].

The prevalence of eating disorders this study was similar to those of other contemporary studies of 12 month or current prevalence, but higher than those of the New Zealand population based study [20]. Although the latter study reported a low 12 month prevalence of eating disorders: anorexia nervosa <0.1% (95% C.I. 0.0–0.1), bulimia nervosa 0.4% (0.3–0.6) and any eating disorder 0.5% (0.3–0.6) the study did not report on rates of binge eating disorder or other EDNOS diagnoses, the most common of all eating disorders in clinical settings [27]. The recent National US survey [19] assessed eating disorder symptoms more broadly. Hudson *et al* [19] found a similar 12 month prevalence of bulimia nervosa to the New Zealand study and to the present study, and a similar prevalence of binge eating disorder to the present study.

The findings of an increase in behaviors is also consistent with Hudson *et al* [19] finding of cohort effects for bulimia nervosa, binge eating disorder and any binge eating, behaviors that, as in the present study, occurred in both men and women. Fichter *et al* [21] investigated secular trends in eating disorder symptoms over 18 years in two cross-sectional surveys and found mixed results with differing effects by site, time and gender. For all participants total Anorexia Nervosa Inventory for Self-rating [28] scores of ‘feelings of insufficiency’ and ‘bulimic behavior’ significantly decreased while ‘negative effects of meals’ (nausea, fullness, uneasiness and vomiting) increased. Severe bulimic behavior increased over time in females in Munich. While binge eating is a common problem in the overweight and obese [29], the increase in bulimic behaviors remained significant when controlling for secular trends and increasing weight in the population samples. Other putative factors that may have contributed include increasing weight concern or an increase in other risk factors for eating disorders, and future studies should study weight and shape concerns concomitantly with eating disorder behaviors.

When compared to the general population of South Australia (aged 15 years or older) according to the Australian Bureau of Statistics census [30] age increased by two years (from 49 to 51 years) between 1996 and 2006. The participants of our study were on average two years older in 2005 compared to 1995 (45 to 43 years; adjusted for cluster sampling). The sub-group of binge eating respondents were also on average 2 years older in the 2005 survey and the difference between the cross-sections was not significant. However people who reported purging and dieting were on average 10 years older in the second survey, a difference much greater than expected by population ageing. The finding was unexpected and needs to be replicated with a third cross-sectional survey that also assesses age of onset of the behaviors. If a true finding a possible explanation is that these are behaviors that are associated with a now older birth cohort. A recent Canadian general population study of ‘weight preoccupied women’ [31] has also found that, after controlling for self-perceived weight, self-esteem and relevant socio-demographic characteristics, women in the 45–64 year age group were more likely to be ‘food preoccupied’ (e.g. bingeing, feeling guilty after eating, feeling ‘food controls your like’, and giving too much time and thought to food) than those aged less than 45 years. At the least, the findings are consistent with emerging literature [32] that indicates that mid aged and older people also suffer from eating disorders.

The main conclusion from the present study is that eating disorders appear to be increasing in point prevalence in Australia, but this increase may be in EDNOS rather than anorexia nervosa or bulimia nervosa. The findings are consistent with a true increase in incidence, particularly as there is no evidence to our knowledge of a recent change in the natural history and/or efficacy of treatments for EDNOS. The present study cannot explain why there should have been such an increase. However, we speculate that the rising tide of public concern over the (also very real) increase in weight disorder in the general population in Australia, may have contributed to increased use of weight control behaviors, and to binge eating as a consequence of dietary restriction, to a less intense and/or severe degree than is seen in bulimia nervosa where these behaviors underpin the theoretical model of development and maintenance of symptoms [33]. Finally, the finding of significant burden as reflected in the high frequency of ‘days out of role’ associated with EDNOS [23] indicates that this is not a trivial problem, and of the eating disorders, these disorders are also those most associated with obesity and weight disorder, in itself of major public health importance. The results also have implications for classification schemes (e.g. the DSM-IV [6]) which provide poor definition of EDNOS, and improvement is needed to facilitate further research and understanding of this growing problem.
